# Teaching Games for Understanding in Game Performance and Psychosocial Variables: Systematic Review and Meta-Analysis of Randomized Control Trial

**DOI:** 10.3390/children10030573

**Published:** 2023-03-17

**Authors:** Marcos Ortiz, Lourdes Meroño, María T. Morales-Belando, Raquel Vaquero-Cristóbal, Noelia González-Gálvez

**Affiliations:** Facultad de Deporte., UCAM Universidad Católica de Murcia, 30107 Murcia, Spainrvaquero@ucam.edu (R.V.-C.);

**Keywords:** models-based practice, modified games, pedagogical approaches, pedagogical models, physical education, sports pedagogy

## Abstract

Different authors have reported on the influence of the Teaching Games for Understanding (TGfU) intervention on game performance and psychosocial variables. This review aimed: (a) to explore the TGfU experimental studies; (b) to compare the effects of the TGfU vs. technical approach pedagogy on game performance; and (c) to determine the effect of the TGfU approach on game performance and psychosocial variables (motivational climate, task orientation, perceived competence and enjoyment). This systematic review with meta-analysis adheres to the Preferred Reporting Items for Systematic Reviews and Meta-Analyses (PRISMA) guidelines. Four databases (PubMed, WOS, EBSCO and Google scholar metasearch) were searched. Study quality was measured with the Physiotherapy Evidence Database (PEDro) score. Thirteen studies were included. A pooled analysis of all interventions demonstrated a large significant improvement in decision making for TGfU when compared to technical approach pedagogy (SMD = 5.93, I2 = 98%; CI (95%) = 2.15–9.71; *p* = 0.004) and no differences between groups for skill execution (SMD = 1.70; I2 = 8%; CI (95%) = −5.34–8.73; *p* = 0.60). The effect of a TGfU intervention on game performance is strong (decision making, execution skills and tactical skills). Moderate evidence is reported by psychosocial variables (motivational climate, task orientation, perception of motivation and achievement in physical education). In addition, it is unclear its effect on perceived competence, enjoyment, knowledge of the game and intention to be physically active. TGfU intervention could be an appropriate approach for males and females in the context of education or sport. There is a need for a greater number of studies.

## 1. Introduction

Teaching Games for Understanding (TGfU) is a Game-Based Approach [1] that “advocates learner playing the game as the central organizational feature of a lesson” [2]. TGfU integrates tactics and skills into the games [3,4]. Bunker and Thorpe [5] argued that some categories of sports show similar tactical strategies, and therefore, they suggested that games could be used to teach the main tactics required for each game by following four pedagogical principles: sampling (use of modified games and sport facilitating the games’ integration); exaggeration (change game structures to promote and exaggerate a particular aspect of the game); representation (small-sided modified games structured to suit the age and/or experience of the players); questioning (pose questions to promote problem solving in students, i.e., what to do, when to do it and why to do it) [5]. The TGfU approach proposes the use of games, as they facilitate the overcoming of limitations by placing the learning of skills in a specific context, thus allowing the understanding of games, the development of tactical knowledge and the improvement of problem-solving abilities through the execution of skills and decision-making actions [5,6]. This approach follows six-steps: (1) game form, (2) game appreciation, (3) tactical awareness, (4) decision making, (5) skill execution and (6) performance.

Since its origin, multiple variables have been developed around the world in order to fulfil particular needs according to social and cultural contexts. Two such examples are the Tactical Games Model, created in the United States, or Game Sense, produced in Australia. The first focused on the coach’s tactical approach, while the second is centered on young athletes’ training [7,8]. This approach influenced the development of other proposals such as the Play Practice [9] competition model of sports games, the progressive approach to play for the teaching of volleyball [10], the Games Concept Approach, Ballschule, or the Tactical Decision Learning Model [2,11]. Although these variants have a common reference in TGfU, proposed by Bunker and Thorpe, they present some differences from it. For instance, Tactical Games simplified the six-step teaching of TGfU into a three-step cycle; Game Sense was a deviation from the six-step learning cycle of TGfU, in which the appreciation of the game of play occurs prior to the development of the technique; and Play Practice does not emphasize the development of “thinking players” through guided discovery using questions as a pedagogical tool [12]. Rink et al. [13], after their review which included different Game-Based Approaches, indicate that the results are inconclusive, probably due to the inclusion of, among others, different types of Game-Based Approaches; and therefore, as Stolz et al. [12] claimed, the challenge of TGfU meta-analysis lies in the differentiation in the Game-Based Approach variants.

Given the growing application of the TGfU approach in teaching–learning contexts and its benefits, the studies focused on this model are numerous. In general, the literature discourse favors the TGfU approach to improve educational variables such as decision making, tactical awareness, technical improvement, motivation towards the sessions and increased motor engagement time [14,15,16,17,18,19,20]. The most studied variables are decision making [21,22,23,24,25,26] and execution skills [23,24,26,27,28,29,30,31]. In addition, other variables have been analyzed, such as: tactical skills [21,22,23,25,26,29,32], knowledge of the game [15,26], psychosocial variables [23,26,32,33] and academic result [33]. These investigations apply intervention programs in different contexts (school or club), years, genders, sports, duration and frequency, aspects that could have an influence on the results. In addition, some empirical studies have tried to compare TGfU vs. other pedagogical approaches in order to conclude which approach is more appropriately developing these variables in different contexts, such as in the context of physical education or in sport [15,21,24]. However, to date, few systematic reviews about interventions based on the TGfU approach have been conducted. Initially, Oslin and Mitchell [2] conducted a primary review around five common objectives of physical education and sport programs. Seven years later, Harvey and Jarrett [34] did so to offer guidelines and recommendations set out for future studies designed based on the TGfU approach. However, Stolz and Pill [12] highlighted the disparity between researchers and teachers applying the approach.

Recently, some reviews focused on the TGfU approach have also been conducted but with different aims than the present research. González-Víllora et al. [35] assessed hybridizations conducted among pedagogical models, including the TGfU approach. Kinnerk et al. [36] reviewed papers focused on coaching in competitive team sport settings. Barba et al. [37] conducted a systematic review of the research on TGfU in physical education over a six-year period (2014–2019). The results showed that according to the aim of studies based on the TGfU approach, cognitive learning is the most frequently assessed (focusing on improvement of game development, such as tactical aspects, decision making, technical skills or level of physical activity), leaving motor skills, physical abilities and body expression underrepresented. Among these, Abad et al. [38] developed a recent systematic review with meta-analysis that studied the effect of teaching games. Nevertheless, this study included all the Game-Based Approaches (Teaching Games for Understanding, Tactical Games Approach, Technical–tactical model with an emphasis on orientation to tactical, Teaching Games for Understand revised and Game approach) and only analyzed two variables (decision making and skill execution). Authors such as Morales-Belando et al. [39] also developed a systematic review of TGfU studies, but they did so from a practice-referenced perspective to explore how TGfU researchers designed their interventions based on the teaching–learning implementation features (intervention design as a function of the context, intervention length, lesson content, basic lesson elements, lesson alignment, teacher/coach experience with the approach, and lesson validation and treatment verification) and their association with learners’ outcomes. The results found that studies on TGfU measured and reported learners’ outcomes in a variety of ways, being difficult to draw clear conclusions about the relationships between the variables of the teaching–learning process. The previous reviews did not clarify if physical/psychomotor, cognitive and affective/social development can be fostered via TGfU through experimental articles with a control group. In addition, it is necessary to know the effect of the approach as an intervention method from an educational and coaching perspective.

The variables and diversity of research have increased exponentially in recent years, which calls for a more exhaustive analysis. An up-to-date understanding according to the effect of the TGfU approach on teaching–learning variables may provide further guidance to design as a function of the context and alignment of their elements. Therefore, the aims of this systematic review and meta-analysis were: (a) to explore the TGfU experimental studies; (b) to compare the effects of the TGfU vs. technical approach pedagogy on game performance; and (c) to determine the effect of the TGfU approach on game performance and psychosocial variables (motivational climate, task orientation, perceived competence and enjoyment).

## 2. Materials and Methods

### 2.1. Study Design

To carry out the present systematic review with meta-analysis, the guidelines of the Preferred Reporting Items for Systematic reviews and Meta-Analyses (PRISMA) were followed [40,41].

### 2.2. Study Selection

The inclusion criteria were: (a) articles that explicitly indicate the use of TGfU as an intervention method in the method section, (b) articles written in English, Spanish and/or Portuguese, and (c) experimental articles with a control group. The exclusion criteria were: (a) articles that indicate the use of other Game-Based teaching (such as Tactical Games Model, Game Sense, competition model of sports games, Games Concept Approach and Tactical Decision Learning Model) instead of TGfU approach, or (b) books, pedagogical papers, congresses, systematic reviews and theoretical papers.

### 2.3. Study Strategies

Two reviewers independently performed a literature search. The systematic search was performed using different databases: PubMed, Web of Science, EBSCO and the Google Scholar meta-search. These databases were selected because they included PE articles published in journals indexed in the Journal Citation Report (JCR) or a similar one (e.g., the Scimago Journal Rank-SJR). This search encompassed all the experimental studies conducted up to 1 April 2021. The year of publication was not limited. The search was conducted from 1 October 2020 to 1 April 2021 The following search terms and MeSH terms were used: TGfU, sport pedagogy, tactical games approach, tactical games model, games centered approach, game sense approach, games-based approach, games teaching, Bunker and Thorpe; along with other words: intervention, experimental, quasi experimental and randomized controlled trial. Additionally, the English Boolean data types ‘and’ and ‘or’ were used.

### 2.4. Data Collection and Synthesis

The articles found were independently reviewed by two authors following PRISMA instructions. If there was any disagreement, the search was carried out again [41]. Cohen’s Kappa was used to calculate the reliability between the two authors, finding a high level of agreement (Kappa = 0.89) [42]. The authors collected data from the design of the study, characteristics of the participants (number, gender, and age), characteristics of the intervention (scope, sport, session/week, minutes/session, duration), interventions with control group or without control group and measure variables [41].

### 2.5. Assessment of Risk of Bias

To discover the quality of the different studies, the score established by the “Physiotherapy Evidence Database” (PEDro) scale was used [43]. PEDro provides the researcher with a series of details which indicate the excellence of the articles, showing which are valid for quality analysis and studies [43]. Table 1 shows the score of the PEDro scale for each of the items included. Criterion number 1 identifies the external validity, and it is not used to calculate the PEDro score. Each criterion is explained in Table 1, and it is scored as yes (1 point) or no (0 points). The total score is the sum of every item (except the first). The results show a quality between 4 and 9 points with an average of 7.38 points; thus, it was considered as acceptable. To show the meta-analysis without the risk of contamination due to the quality of the study.

### 2.6. Statistical Analysis

The meta-analysis was performed with the R software version 3.6.0. Copyright (C) 2019 (R Foundation for Statistical Computing). Meta-analysis was completed for continuous data using the change in mean (M) and standard deviation (SD) between baseline and final (pre–post intervention) In this case, only research studies with a control group (CG) and an experimental group (EG) were considered. The studies were grouped according to the assessment variable: game performance (decision making: five studies; and skill execution: three studies). It was not possible to perform the meta-analyses with psychosocial variables due to the disparity in methodologies and results. For studies that did not have the necessary data, SD were calculated and imputed when possible, using standard errors (SE) and confidence intervals (CI). The DerSimonian–Laird (Cohen) pooling method was used and heterogeneity was assessed, using the Cochrane Q test (Chi^2^), Higgins I^2^ and significance (*p*), to determine the appropriateness of the application of a fixed or random effect model for the pooled analysis [46]. Studies were weighted according to sample within and between studies. A pooled summary mean and 95% CI were calculated for subgroups (article) in order to group the data into the groups. Random models using the Restricted Maximum Likelihood Method (REML) were utilized. The heterogeneity was measured using the I2 statistic, considering a high heterogeneity if I2 ≥ 75% [47].

## 3. Results

**Figure 1:**
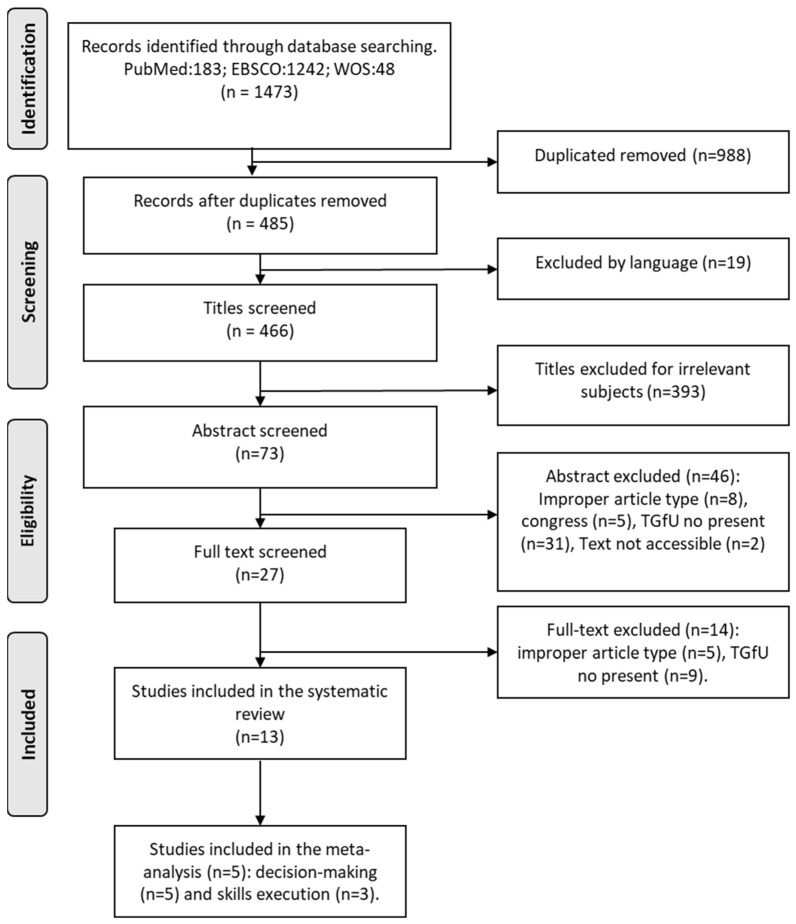
Flow Diagram of searched, screened, and included studies.

A total of 1473 publications were obtained in the first instance. Finally, 13 studies were included in this review, and 5 were included in meta-analysis (decision-making variable (n = 5) and skills execution (n = 3) (Figure 1). The characteristics of the included studies are shown in Table 2.

These studies included a total of 468 cases and 366 controls. The average sample size was 64.3 (range 20–237). Eight studies were conducted with both men and women [23,24,26,27,31,32,48], one with only women [29], one with only men [22] and two other studies where gender was not specified [21,25].

In the studies where women and men were included, in four of these studies, the participation was mostly women, with 54.89%, 62.06%, 52.17%, 73.68% and 54.9% [26,48,49], respectively; in three studies, participation was mostly men, 66.7%, 58.3% and 56.09% [23,33], respectively; while in one study, participation was the same between both genders [24].

Of the 13 studies, 5 studies (38.46%) carried out their intervention at high schools or sports clubs [22,25,26,27,50], 3 were carried out in primary education (23.07%) [23,44,48], 3 were carried out in secondary education (23.07%) [31,32,33], 1 study was at elementary and high schools (7.69%) [29] and 1 study was developed at university (7.69%) [21].

The average duration of the programs was 6.27 weeks (range 1–12). The average time used for each session was 59.66 min (range 20–150). The average number of sessions per week was 2.83 (range 2–9). The average number of sessions over the duration of the intervention was 13.76 (range 6–24).

In terms of inclusion criteria, three studies did not show the participants to any type of inclusion or exclusion criteria [21,33,44]. One study indicated female gender [29] and the other indicated male gender [22]. Another criteria was experience, finding that it ranged from those who had no experience in the sport to be practiced [24,27,49] or who even had some experience [25,26].

Regarding game performance, a total of nine studies reported results for decision making, after implementing a TGfU intervention. Six studies showed significant improvements in the intervention with a TGfU program [21,22,23,24,25,26], one study showed improvements in two out of the three groups [29] or in same variables [45]. Of these, only five studies presented an experimental group, so only five studies are included in the meta-analysis. Seven studies assessed tactical skills, seven assessed “performance and participation” [21,22,23,25,26,29,32] and three assessed “movement in the field”. Four of them showed improvement after implementation of a TGfU program [23,26,29,32], and the remaining three studies showed improvements in some of the variables of tactical skills [21,22,25]. Of the seven studies, three are those that present a control group and are therefore taken into account for the meta-analysis. Three studies implemented a TGfU intervention and assessed knowledge of the game. One of these studies showed significant improvements in this variable [31], one study reported significant improvements on some of the variables recorded [26], and the last one showed no improvement [44].

Some studies assessed the effect of TGfU on psychosocial variables. Most of them found significant improvements in motivational climate [32,33], task orientation [32] and enjoyment [23]. There were just two studies that did not show improvements in perceived competence [26] and enjoyment [26]. Other authors have been interested in its effect on physical activity level. One study shows an improvement in the intention to continue practicing the sport after the intervention [23] in floorball, while another study did not show improvements [26] in sailing. Hortigüela and Garijo [33] reported an improvement in motivation and achievement after a TGfU intervention, which were not observed after a technical approach intervention.

Table 3 and Table 4 show all the studies about game performance that implemented a TGfU approach intervention in the experimental group and a technical approach in the control group. The studies that were not included had no control group. These tables include all cases where decision making (Table 3) and skills execution (Table 4) were assessed. For example, Ashraf [21] presents six cases due to valuing decision making by means of six variables. These tables indicate the sample size, the pre–post test mean change and the standard deviation of each group (TGfU or technical approach) of each variable (decision making or skill execution). A positive SMD indicated a better change in the variable (decision making or skill execution) in the TGfU than the technical approach. For example, Ashraf [21]-1 shows that the group that received a TGfU intervention improved significantly more (SMD = 10.01) in decision making than the group that received a technical approach (*p* ˂ 0.001). In connection with this, in 15 of the 17 cases, TGfU showed better improvements than the technical approach in decision making with statistical significance. This indicates that in most cases, the TGfU approach is presented as a better approach to improving decision making than a technical approach. In connection with skill execution, four cases were in favor of technical approach, four cases were in favor of TGfU, and two cases did not show significant differences between the TGfU or technical approach. These results suggest that there is no difference in the application of one approach or another on skills execution.

**Figure 2:**
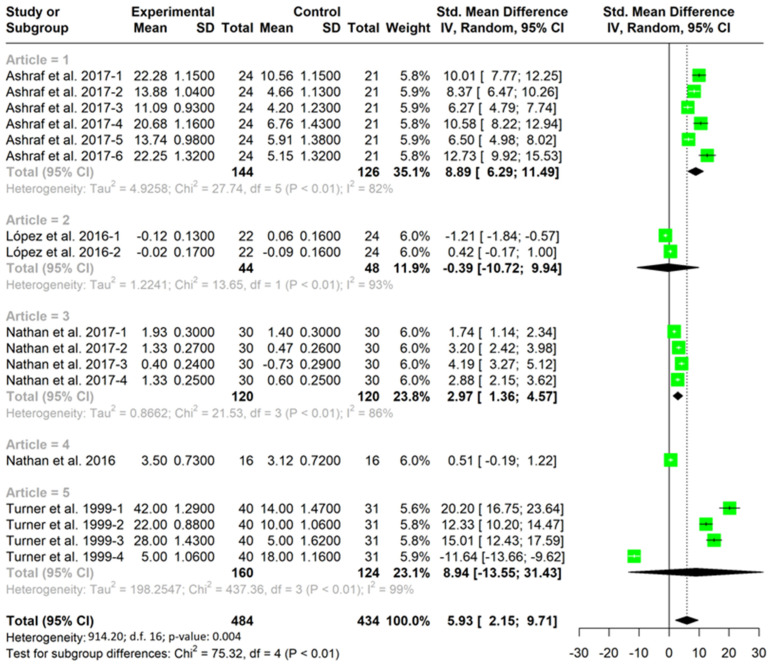
TGfU versus technical approach for improved decision making [21,24,25,31,45].

**Figure 3:**
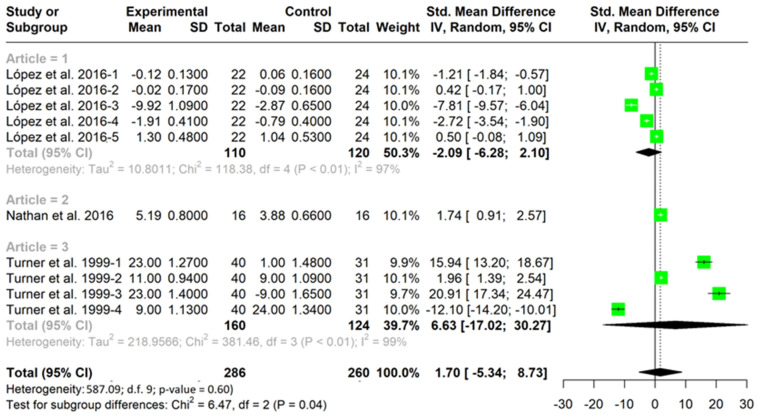
TGfU versus technical approach for technique improvement [25,31,45].

Figure 2 and Figure 3 show the meta-analysis grouped according to studies. Each figure indicates the results for each of the variables analyzed within each study for both decision making (Figure 2) and skill execution (Figure 3). In addition, it includes the mean for each study, as well as the total mean for each variable.

Pooled analysis of all interventions demonstrated a large and significant improvement in decision making for TGfU when compared to technical approach (SMD = 5.93, I2 = 98%; CI (95%) = 2.15–9.71; *p* = 0.004) (Figure 2). This aspect indicates that the TGfU approach is better than the technical approach for improving decision making in the studies analyzed. Pooled analysis for skill execution demonstrated no significant differences between groups in execution skills between TGfU and technical approach (SMD = 1.70; I2 = 8%; CI (95%) = −5.34–8.73; *p* = 0.60), indicating that both techniques are equally valid for the improvement of the skills execution (Figure 3).

The pooled analysis, including only those studies with a score of 7 or higher on the PEDRO scale, report similar results to the previous ones (decision making: SMD = 4.82, CI (95%) = 3.24–6.39; *p* < 0.001; skill execution: SMD = −1.36; CI (95%) = −3.01–0.29; *p* = 0.106).

## 4. Discussion

The first objective of the present systematic review with meta-analysis was to explore the TGfU experimental studies. The results found suggest that TGfU could be an appropriate pedagogical approach to be used in every situation to improve decision making and skill performance, given that the research found shows positive results in both genders and in different settings, although with a certain predominance of the female gender, and there was a greater presence of research in secondary schools or sports clubs, followed by primary school. There were no differences in the efficacy of TGfU, considering the context (university, high school, elementary school or sport club), age or gender. However, it would be necessary to take into account the differences that occur between the school and sports context such as the pedagogical training of the instructors, which could influence the viability of its use [22]. Those differences could be due to researchers, coaches or teachers needing to follow a guide for implementing TGfU [39]. The programs implemented have an approximate duration of 6 weeks, 2–3 times a week and 55 min per session. This favors their inclusion within the context of education and sport. This summary is relevant to teachers and coaches in terms of the recommended duration and frequency. However, the implementation of the approach could be carried out in too short a time to achieve significant outcomes [37]. Arias-Estero et al. [51] concluded that the amount of practice should not be considered as the only variable in the design of interventions with the approach.

The second purpose of the present study was to compare the effect of an TGfU vs. technical approach pedagogy in game performance. The present results are in line with the systemic review with previous meta-analyses of Abad et al. [38]. These authors indicated that, as per the present systematic review with meta-analysis, the tactical approach showed higher improvements in decision making than the technical approach. The study by Abad et al. [38] showed a total effect size of 0.89 (*p* = 0.020) vs. the present meta-analysis, which showed a total effect size of 5.75 (*p* ˂ 0.001). Taking in to account that the Abad et al. [38] study included in their meta-analysis other GBAs in addition to TGfU, this result could indicate that TGfU may be more beneficial than other methodological approaches that focus on tactics for improved decision making (such as Tactical Games Model, Game Sense, competition model of sports games, Games Concept Approach or Tactical Decision Learning Model). However, this aspect is difficult to analyze because the implementation of an approach may depend on the teacher or coach rather than the approach used [52]. Original research studies that compare different GBA could be necessary. In this sense, several researchers have highlighted that in pedagogical approaches focused on tactics, there was a greater transfer between sports, since there are many similarities between them, and they can be used to facilitate learning [11].

In connection with skill execution, the study performed by Abad et al. [38] showed similar result. This study indicated an overall effect size of 0.89 (*p* = 0.190), which is similar to our result (SMD = 1.70; *p* = 0.60). Two of three studies included in the meta-analysis for decision making showed greater improvements in the TGfU group than the technical approach group. No studies reported greater improvements in the technical approach group than in the TGfU group. The TGfU pedagogical approach integrates tactics and skills into games. This does not mean that in the TGfU approach, the execution of the technique is neglected, but that it is developed after understanding the strategies and tactics of the game. Therefore, this meta-analysis suggests that the TGfU approach could be more beneficial than the technical approach because not only did it result in improved skill execution but also improved decision making [53]. In this sense, the TGfU approach is presented as an effective approach to be used by both teachers and coaches to improve both technique and tactics in schoolchildren or athletes of both genders.

The last aim of the present systematic review was to determine the effect of the TGfU approach on game performance and psychosocial variables.

### 4.1. Game Performance

All the studies founded significant improvements in decision making after an intervention with the TGfU approach [21,22,23,24,25,26,31]. Despite the heterogeneity between the studies in relation to age, gender, context and sport, all studies showed improvements. This is a great challenge for teachers and coaches of different educational stages and sports. TGfU is positioned as a valid approach to be implemented to achieve improvements in decision making in those sports in which this mechanism is important. Furthermore, considering the results reported by the meta-analysis, TGfU is positioned as a better approach than a technical approach for its development.

Regarding skill execution, all studies showed improvement after a TGfU approach intervention [22,24,26,29,31]. Taking into account the results previously reported by the meta-analysis, TGfU is not only effective in improving skill execution, but it is also as effective as a technical approach. Bearing in mind that when the technical approach is applied, teachers or coaches spend more time teaching the isolated technique. According to Barakat et al. [54], the acquisition of motor skills is generally carried out in several phases of learning. It is likely a longer intervention time results from a greater learning of skill execution in the TGfU approach group. Therefore, it is necessary more studies to implemented different intervention duration.

Seven studies assessed tactical skills, seven assessed “performance and participation” [21,22,23,25,26,29,32] and three assessed “movement in the field”. Four of them showed improvement after implementation of a TGfU program [23,26,29,32] and the remaining three studies showed improvements in some of the variables of tactical skills [21,22,25]. These results were to be expected since TGfU was designed to promote improvement in performance and participation [5], and game performance is the result of the quality of decision making and skill execution [22]. However, Harvey et al. [22] indicated that the improvement in game performance variables was significant only for first-year participants. This aspect could mean that the choice of approach in the initiation stage is of great relevance. Considering that greater task ability is related to better self-concept and motivation to practice [55], the application of the TGfU approach in the initial stages becomes important.

Three studies implemented a TGfU intervention and assessed knowledge of the game. Two of these studies showed significant improvements in this variable [31,44], and one study reported significant improvements in some of the variables recorded [26]. Game knowledge is an important aspect for TGfU, as it can promote intrinsic motivation [12]. Morales-Belando and Arias-Estero [26] indicate that its participants may not have improved in all variables in their knowledge of sports because they applied the intervention in an extracurricular context, and the participants had previous knowledge of the sport. In this sense, it seems that a comprehensive approach to gaming could be relevant mainly to favor cognitive dissonance in learning games, reflect on practice and favor learning the essentials of gaming and its tactical principles. This is one of the basic pillars of the GBA and represents one of the most difficult processes in sports initiation [56]. It could be argued that the TGfU approach is effective for the knowledge of sport in sports initiation, but that it does not differ from it in later stages; however, more research on this is necessary.

### 4.2. Psychosocial Variables (Motivational Climate, Task Orientation, Perceived Competence and Enjoyment)

Most of the studies in this review found significant improvements in motivational climate [32,33], task orientation [32] and enjoyment [23]. In this sense, Jones et al. [57] indicated that the implementation of TGfU vs. a traditional skills-based approach promoted an improvement in fun and enjoyment and that the students felt an increased environment of autonomy. The learning of sports could be developed in an optimal and holistic, affective, cognitive and physical environment [58]. Indeed, it is known that a traditional approach in which the student does not perceive enjoyment is connected to the student feeling amotivated [59]. A low perceived competence could worsen this aspect in the traditional pedagogical approach. Therefore, when the participants are engaged and the TGfU approach is applied, competence may be improved [60].

One study showed an improvement in the intention to continue practicing the sport after the intervention [23] in floorball, while another study did not show improvements [26] in sailing. The difference between these two studies could be due to the sports being different. Other authors have shown that when the games are included in the session, the moderate–vigorous physical activity (MVPA) is higher than with other times [61]. In addition, MVPA has been reported to be higher in participants with a high physical self-efficacy perception [61]. The TGfU approach improved the perception of competence [60], and therefore, it is suggested that MVPA during the lesson could be increased with this pedagogical approach.

Hortigüela and Garijo [33] reported an improvement in motivation and achievement after a TGfU intervention, which were not observed after a technical approach intervention. This could be explained because physical activity produces physiological changes by increasing serotonin levels [62]. A high serotonin level has been associated with better academic achievement [63]. However, it has also been suggested that not all physical activity has the same effect on serotonin levels. Aerobic performance significantly improved serotonin levels, whereas no improvements for anaerobic or strength physical activity have been observed. This could be the explanation for these results [64].

The present study is not without a limitation. Although publication bias was not identified, the analysis showed moderate to high heterogeneity. This could be because the formulas used to calculate the standard deviation of the change of the angle, when not provided in the article, provide conservative results and reports of a high standard deviation, which could influence heterogeneity. In addition, the small number of studies included in the meta-analysis could explain the high heterogeneity [65]. Furthermore, it is necessary to performance more randomized controlled trial to support these results.

## 5. Conclusions

TGfU intervention could be an appropriate approach for males and females in the context of education or sport. There is great feasibility for TGfU to be applied both in sports or teaching–learning contexts.

The application of the TGfU approach showed significantly greater improvements in decision making and the same improvements in skills execution as a traditional model. This is a great contribution to the teacher and the coach, and the TGfU approach is recommended instead of the technical approach since both achieved the same learning outcome with the technique, and TGfU is also better for learning decision making.

There was strong evidence for the effect of the TGfU approach on game performance (decision making, skill execution and tactical skills) and moderate evidence on psychosocial variables (motivational climate, task orientation, perception of motivation and achievement in physical education); the effect on other psychosocial variables such as perceived competence, enjoyment, knowledge of the game and intention to be physically active remains unclear. There is a need for a greater number of studies that include a random control group, with analysis performed separately by gender, that include a wide range of variables, and that compare different durations and the frequency of the intervention, to test the effect of TGfU between gender and different contexts and to determine the appropriate frequency and duration. 

## Figures and Tables

**Table 1:** Risk of bias according to the PEDro Scale.

Study	1	2	3	4	5	6	7	8	9	10	11	Total Score
Ashraf 2017 [21]	Y	Y	N	Y	Y	N	N	Y	Y	Y	Y	7
Calabria-Lopes et al., 2019 [27]	Y	Y	Y	N	Y	N	Y	Y	N	N	Y	6
Chiva-Bartoll et al., 2018 [32]	Y	Y	Y	N	Y	N	N	Y	Y	Y	Y	7
Harvey et al., 2010 [28]	Y	Y	Y	N	Y	N	Y	Y	Y	N	Y	7
Hortigüela Alcalá et al., 2017 [33]	Y	Y	Y	N	Y	N	Y	Y	Y	Y	Y	8
López et al., 2016 [31]	Y	Y	Y	Y	Y	N	Y	Y	Y	Y	Y	9
Morales-Belando et al., 2018 [23]	Y	Y	Y	Y	N	N	Y	Y	Y	Y	Y	8
Morales-Belando et al., 2017 [26]	Y	Y	Y	Y	Y	N	N	Y	Y	Y	Y	8
Nathan 2017 [25]	Y	Y	Y	Y	Y	Y	N	Y	Y	Y	Y	9
Nathan 2016 [24]	N	Y	Y	Y	Y	N	N	Y	Y	Y	Y	8
Olosová et al., 2015 [44]	Y	Y	Y	Y	N	N	N	Y	Y	Y	Y	7
Turner et al., 1999 [45]	N	Y	N	N	N	N	N	Y	N	Y	Y	4
Žuffová et al., 2015 [29]	Y	Y	Y	Y	N	N	Y	Y	Y	Y	Y	8

Y = Yes; N = No; 1 = eligibility criteria were specified; 2 = subjects were randomly allocated to groups (in a crossover study, subjects were randomly allocated an order in which treatments were received); 3 = allocation was concealed; 4 = the groups were similar at baseline regarding the most important prognostic indicators; 5 = there was blinding of all subjects; 6 = there was blinding by those responsible for the intervention; 7 = there was blinding of all assessors who measured at least one key outcome; 8 = measures of at least one key outcome were obtained from more than 85% of the subjects initially allocated to groups; 9 = all subjects for whom outcome measures were available carried out the intervention or control condition as allocated or, where this was not the case, data for at least one key outcome was analyzed by “intention to do the intervention”; 10 = the results of between-group statistical comparisons are reported for at least one key outcome; 11 = the study provides both point measures and measures of variability for at least one key outcome; total score: each satisfied item (except the first) adds 1 point to the total score.

**Table 2:** Characteristics of the studies included in the systematic review.

Study	Design/Sample	Age/Scope/Sport	Time	Criteria	Intervention	Results	SR/MA
Ashraf 2017 [21]	Random/*n* = 45 EG = 24; CG = 21	Age = 20: EG = 20 ± 1.9, CG = 20 ± 1.2 College/football	10 weeks	-	EG = TGfU CG = TA	TGfU: + DM	MA
Calabria-Lopes et al., 2019 [27]	Pre-experimental/*n* = 20/18; EG = 18 EG_Male_ = 66.7%	Age = 9–12 years *M* = 10.89 ± 1.02 Sport club/basketball	4.5 ses/week 5 h/day 9 ses	EC = less than 75% attendance	EG = TGfU	TGfU: + SE	SR
Chiva-Bartoll et al., 2018 [32]	Quasi-experimental *n* = 96; EG = 31; CG = 65; EG_Male_ = 15; EG_Female_ = 16; CG_Male_ = 31; CG_Female_ = 34	Age = 15–16 *M* = 15 High school/handball	2 ses/week 45 min/ses 8 weeks	IC = fourth grade of secondary	EG = TGfU. CG = TA	TGfU: + motivational climate	SR
Harvey et al., 2010 [22]	Pre-experimental *n* = 34	Age = 14–18 Sport club of Football	45–60 min/ses 12 weeks	IC = male sex.	EG = TGfU	TGfU: + DM and SE	SR
Hortigüela Alcalá and Garijo 2017 [33]	Quasi-experimental *n* = 237; EG = 128; CG = 109; Male = 58.3%	Age = 12–17 years *M* = 13.32 ± 2.31 High school/basketball	2 ses/weeks; 60 min/ses 12 weeks 24 ses	-	EG = TGfU CG = TA	TGfU: + Perceptions of motivation and achievement	SR
López et al., 2016 [31]	Quasi-experimental *n* = 46; EG = 22; CG = 24; EG_Male_ = 10; EG_Female_ = 12; CG_Male_ = 12; CG_Female_ = 12	Age = 14–15 High school/basketball	2 ses/week 45 min/ses 4.5 weeks 9 ses	IC = be inexperienced in the sport	EG = TGfU CG = TA	TGfU: + procedural knowledge, skill execution in isolation, SE in a real-game situation. No: + DM	MA
Morales-Belando et al., 2018 [23]	Pre-experimental *n* = 41; EG = 41; EG_Male_ = 23; EG_Female_ = 18	Age = 11–12 *M* = 11.73 ± 0.66 Elementary school/floorball	2 ses/week 55 min/ses 3 weeks	IC = same location	EG = TGfU	TGfU: + DM, SE, cover, support, game performance, participation, enjoyment, perceived competence and intention to be physically active	SR
Morales-Belando et al., 2017 [26]	Pre-experimental *n* = 19; EG_Male_ = 5; EG_Female_ = 14	Age = 7–10 years *M* = 8.44 Sport club/sailing	2 ses/week 60 min/ses 5 weeks 12 ses	EC = miss more than 3 sessions	EG = TGfU CG = TA	TGfU: + DM, SE, knowledge, cover, support, game performance, participation, enjoyment, perceived competence no +: intention to be physically active	SR
Nathan 2017 [25]	*n* = 60 Malaysia Random: EG = 15; CG = 15; Indian Random: EG = 15; CG = 15	Age = 14–16 years *M* = 15 ± 1.03 Sport club/hockey	3 ses/week 120 min/ses 5 sessions	IC = 3 experience years in the sport	EG = TGfU CG = TA	TGfU: + DM and SE	MA
Nathan 2016 [24]	Random *n* = 32; EG = 16; CG = 16 EG_Male_ = 8; EG_Female_ = 8; CG_Male_ = 8; CG_Female_ = 8	Age = 15–16 *M* = 15.5 ± 1.0 Sport club/badminton	2 ses/week 40 min/ses 5 weeks	IC = no experience in sport using TGfU	EG = TGfU CG = TA	TGfU: + DM in some variables, SE, movement ability and SE	MA
Olosová et al., 2015 [44]	Quasi-experimental TS = 69; EG = 29; CG = 40	Age = 10–12 Elementary school/basketball	2 ses/week 45 min/ses 8 weeks	-	EG = TGfU CG = TA	TGfU: + DM, SE and knowledge	SR
Turner et al., 1999 [45]	Random *n* = 71; EG = 40; CG = 31	Age = 10–12 Elementary school/hockey	2 ses/week 45 min/ses 8 weeks	-	EG = TGfU CG = TA	TGfU: + DM in some variables and SE	MA
Žuffová et al., 2015 [29]	Quasi-experimental *n* = 66; EG = 36; CG = 30; EG_Female_1 = 10; EG_Female_2 = 12; EG_Female_3 = 14; CG_Female_1 = 11; CG_Female_2 = 10; CG_Female_ 3 = 9	Age = 11–16; *M* EG_Female_1 = 11.6; *M* EG_Female_2 = 13.8; *M* EG_Female_3 = 15.8; *M* CG_Female_1 = 11.7; *M* CG_Female_2 = 13.8; *M* CG_Female3_ = 15.8; Elementary and high School/ultimate frisbee	2 ses/week 20 min/ses 6 week 12 ses	IC = female sex	EG = TGfU CG = TA	TGfU: + DM in two out of three groups and SE	SR

Note: n = sample size; EG = experimental group; CG = control group; M = mean; ses = session; min = minutes; IC = inclusion criteria; EC = exclusion criteria; TA: Technical approach; + significantly improve; DM = decision making; SE = skill execution; MA = meta-analysis; SR = systematic review.

**Table 3:** Meta-analysis TGfU versus technical approach for improvement in game performance (decision making).

	TGfU			Technical Approach			*SMD*	95% *CI*	z	*p*	Weight (%)
	*n*	*M*	*SD*	*n*	*M*	*SD*					
Ashraf 2017 [21]-1	24	22.28	1.15	21	10.56	1.15	10.01	7.77; 12.26	8.75	˂0.001	5.7
Ashraf 2017 [21]-2	24	13.88	1.04	21	4.66	1.13	8.37	6.46; 10.27	8.62	˂0.001	5.8
Ashraf 2017 [21]-3	24	11.09	0.93	21	4.20	1.23	6.27	4.79; 7.74	8.32	˂0.001	6.0
Ashraf 2017 [21]-4	24	20.68	1.16	21	6.76	1.43	10.58	8.22; 12.94	8.78	˂0.001	5.7
Ashraf 2017 [21]-5	24	13.74	0.98	21	5.91	1.38	6.50	4.98; 8.03	8.37	˂0.001	6.0
Ashraf 2017 [21]-6	24	22.25	1.32	21	5.15	1.32	12.73	9.91; 15.54	8.86	˂0.001	5.5
López et al., 2016 [31]-1	22	−0.12	0.13	24	0.06	0.16	−1.21	−1.84; −0.57	3.74	0.002	6.2
López et al., 2016 [31]-2	22	−0.02	0.17	24	−0.09	0.16	0.42	−0.17; 1.00	1.40	0.162	6.2
Nathan 2017 [25]-1	30	1.93	0.30	30	1.40	0.30	1.74	1.14; 2.34	5.69	˂0.001	6.2
Nathan 2017 [25]-2	30	1.33	0.27	30	0.47	0.26	3.20	2.42; 3.98	8.05	˂0.001	6.1
Nathan 2017 [25]-3	30	0.40	0.24	30	−0.73	0.29	4.19	3.26; 5.12	8.87	˂0.001	6.1
Nathan 2017 [25]-4	30	1.33	0.25	30	0.60	0.25	2.88	2.15; 3.62	7.68	˂0.001	6.1
Nathan 2016 [24]	16	3.50	0.73	16	3.12	0.72	0.51	−0.19; 1.22	1.42	0.155	6.2
Turner et al., 1999 [45]-1	40	42	1.29	31	14.00	1.47	20.20	16.75; 23.65	11.47	˂0.001	5.2
Turner et al., 1999 [45]-2	40	22	0.88	31	10.00	1.06	12.33	10.19; 14.47	11.30	˂0.001	5.8
Turner et al., 1999 [45]-3	40	28	1.43	31	5.00	1.62	15.01	12.43; 17.59	11.39	˂0.001	5.6
Turner et al., 1999 [45]-4	40	5	1.06	31	18.00	1.16	−11.64	−13.67; −9.62	11.27	˂0.001	5.8

TGfU = Teaching Game for Understanding; *n* = sample size; *M* = mean; *SD* = standard deviation; *SMD* = standardized mean difference.

**Table 4:** TGfU versus technical approach meta-analysis for improvement in game performance (skill execution).

	TGfU			Technical Approach			*SMD*	*CI* 95%	*z*	*p*	Weight (%)
	*n*	*M*	*SD*	*n*	*M*	*SD*					
López et al., 2016 [31]-1	22	−0.12	0.13	24	0.06	0.16	−1.21	−1.84; −0.57	3.74	0.002	10.5
López et al., 2016 [31]-2	22	−0.02	0.17	24	−0.09	0.16	0.42	−0.17; 1.00	1.40	0.162	10.5
López et al., 2016 [31]-3	22	−9.92	1.09	24	−2.87	0.65	−7.81	−9.57; −6.04	8.67	<0.001	9.9
López et al., 2016 [31]-4	22	−1.91	0.41	24	−0.79	0.40	−2.72	−3.54; −1.90	6.50	<0.001	10.5
López et al., 2016 [31]-5	22	1.30	0.48	24	1.04	0.53	0.50	−0.08; 1.09	1.68	0.093	10.5
Nathan 2016 [24]	16	5.19	0.80	16	3.88	0.66	1.74	0.91; 2.57	4.12	<0.001	10.4
Turner et al., 2016 [45]-1	40	23.00	1.27	31	1.00	1.48	15.94	13.20; 18.68	11.41	<0.001	9.1
Turner et al., 2016 [45]-2	40	11.00	0.94	31	9.00	1.09	1.96	1.39; 2.54	6.69	<0.001	10.5
Turner et al., 1999 [45]-3	40	23.00	1.40	31	−9.00	1.65	20.91	17.34; 24.48	11.48	<0.001	8.3
Turner et al., 1999 [45]-4	40	9.00	1.13	31	24.00	1.34	−12.10	−14.21; −10.00	11.29	<0.001	9.7

TGfU = Teaching Games for Understanding; *n* = sample size; *M* = mean; *SD* = standard deviation; *SMD* = standardized mean difference.

## Data Availability

The meta-analysis database can be requested from the corresponding author of the article.

## References

[1] (2021). Teaching Games for Understanding Special Interest Group (TGfU SIG). Game-Based Consensus Statement. http://www.tgfu.info/game-based-consensus-statement.html.

[2] Oslin J.M., Mitchell S.A., O’Sullivan M., Kirk D., MacDonald D. (2006). Game-Centered Approachers to Teaching Physical Education. Handbook of Physical Education.

[3] Gutierrez D. (2016). Game-Centered Approaches: Different Perspectives, Same Goals—Working Together for Learning. Res. Q. Exerc. Sport.

[4] Psotta R. (2010). Uplatnění Kognitivního Modelu ve Výuce Fotbalu. Tělesná Výchova A Sport Mládeže.

[5] Bunker D., Thorpe R. (1982). A Model for the Teaching of Games in the Secondary School. Bull. Phys. Educ..

[6] Renshaw I., Araújo D., Button C., Chow J.Y., Davids K., Moy B. (2016). Why the Constraints-Led Approach Is Not Teaching Games for Understanding: A Clarification. Phys. Educ. Sport Pedagog..

[7] Griffin L.L., Patton K., Griffin L., Butler J. (2005). Two Decades of Teaching Games for Understanding: Looking at the Past, Present, and Future. Teaching Games for Understanding: Theory, Research, and Practice.

[8] den Duyn N. (1997). Game Sense: It’s Time to Play. Sport. Coach.

[9] Launder A., Piltz W. (2006). Beyond Understanding to Skilful Play in Games, through Play Practice. J. Phys. Educ. N. Z..

[10] Mesquita I., Tani G., Bento J.O., Petersen R.S. (2006). Ensinar Bem Para Aprender Melhor o Jogo de Voleibol. Pedagogía do Esporte.

[11] Fernández Río J., Calderón A., Alcalá D.H., Pérez Pueyo Á., Cebamanos M.A. (2016). Modelos Pedagógicos En Educación Física: Consideraciones Teórico-Prácticas Para Docentes. Rev. Española Educ. Física Y Deport. REEFD.

[12] Stolz S., Pill S. (2014). Teaching Games and Sport for Understanding: Exploring and Reconsidering Its Relevance in Physical Education. Eur. Phys. Educ. Rev..

[13] Rink J.E., French K.E., Graham K.C. (1996). Implications for Practice and Research. J. Teach. Phys. Educ..

[14] Maxwell J.P., Capio C.M., Masters R.S.W. (2016). Interaction between Motor Ability and Skill Learning in Children: Application of Implicit and Explicit Approaches. Eur. J. Sport Sci..

[15] Memmert D., Furley P. (2016). Teaching Games for Understanding Conference Supplement from the German Sport University. Res. Q. Exerc. Sport.

[16] Slingerland M., Borghouts L. (2014). Differences in Perceived Competence and Physical Activity Levels during Single-Gender Modified Basketball Game Play in Middle School Physical Education. Eur. Phys. Educ. Rev..

[17] Bredekamp S. (1992). What Is “Developmentally Appropriate” and Why Is It Important?. J. Phys. Educ. Recreat. Danc..

[18] Fairclough S.J., Stratton G. (2006). A Review of Physical Activity Levels During Elementary School Physical Education. J. Teach. Phys. Educ..

[19] Fu Y., Gao Z., Hannon J.C., Burns R.D., Brusseau T.A. (2016). Effect of the SPARK Program on Physical Activity, Cardiorespiratory Endurance, and Motivation in Middle-School Students. J. Phys. Act. Health.

[20] Hastie P.A., Trost S.G. (2002). Student Physical Activity Levels During a Season of Sport Education. Pediatr. Exerc. Sci..

[21] Osman A. (2017). Effects of teaching games for understanding on tactical awareness and decision making in soccer for college students. Ovidius Univ. Ann. Ser. Phys. Educ. Sport. Mov. Health.

[22] Harvey S., Cushion C., Wegis H., Massa-Gonzalez A. (2010). Teaching Games for Understanding in American High-School Soccer: A Quantitative Data Analysis Using the Game Performance Assessment Instrument. Phys. Educ. Sport Pedagog..

[23] Morales-Belando M.T., Calderón A., Arias-Estero J.L. (2018). Improvement in Game Performance and Adherence after an Aligned TGfU Floorball Unit in Physical Education. Phys. Educ. Sport Pedagog..

[24] Nathan S. (2016). Badminton Instructional in Malaysian Schools: A Comparative Analysis of TGfU and SDT Pedagogical Models. Springerplus.

[25] Nathan S. (2017). The Effect of Teaching Games of Understanding as a Coaching Instruction Had on Adjust, Cover and Heart Rate among Malaysian and Indian Junior Hockey Players. Sports.

[26] Morales-Belando M.T., Arias-Estero J.L. (2017). Effect of Teaching Races for Understanding in Youth Sailing on Performance, Knowledge, and Adherence. Res. Q. Exerc. Sport.

[27] Calabria-Lopes M., Greco P.J., Perez-Morales J.C. (2019). Teaching Games for Understanding in Basketball Camp: The Impact on Process and Product Performance. Ricyde-Rev. Int. Cienc. Del Deport..

[28] Harvey S., Cushion C., Massa-Gonzalez A. (2010). Learning a New Method: Teaching Games for Understanding in the Coaches’ Eyes. Phys. Educ. Sport Pedagog..

[29] Žuffová Z., Zapletalová L. (2015). Efficiency Of Different Teaching Models In Teaching Of Frisbee Ultimate. Acta Fac. Educ. Phys. Univ. Comen..

[30] Kampf R., Stolero N. (2018). Learning About the Israeli-Palestinian Conflict Through Computerized Simulations: The Case of Global Conflicts. Soc. Sci. Comput. Rev..

[31] Lopez I., Praxedes A., del Villar F. (2016). Effect of an intervention teaching program, based on tgfu model, on the cognitive and execution variables, in the physical education context. Eur. J. Hum. Mov..

[32] Chiva-Bartoll Ó., Salvador-García C., Ruiz-Montero P.J. (2018). Teaching Games for Understanding and Cooperative Learning: Can Their Hybridization Increase Motivational Climate among Physical Education Students?. (Učenje Igara S Razumijevanjem I Surad. Učenje Može Li Njihova Hibridizacija Povećati Motiv. Klimu Na Satima Tjelesne I Zdr. Kult.) Croat. J. Educ. Hrvat. Časopis Za Odgoj. I Obraz..

[33] Hortigüela Alcalá D., Garijo A.H. (2017). Teaching Games for Understanding: A Comprehensive Approach to Promote Student’s Motivation in Physical Education. J. Hum. Kinet..

[34] Harvey S., Jarrett K.A. (2014). Review of the Game-Centred Approaches to Teaching and Coaching Literature since 2006. Phys. Educ. Sport Pedagog..

[35] González-Villora S., Evangelio C., Sierra-Diaz J., Fernandez-Rio J. (2018). Hybridizing Pedagogical Models: A Systematic Review. Eur. Phys. Educ. Rev..

[36] Kinnerk P., Harvey S., MacDonncha C., Lyons M. (2018). A Review of the Game-Based Approaches to Coaching Literature in Competitive Team Sport Settings. Quest.

[37] Barba-Martín R.A., Bores-García D., Hortigüela-Alcalá D., González-Calvo G. (2020). The Application of the Teaching Games for Understanding in Physical Education. Systematic Review of the Last Six Years. Int. J. Environ. Res. Public Health.

[38] Abad Robles M.T., Collado-Mateo D., Fernandez-Espinola C., Castillo Viera E., Gimenez Fuentes-Guerra F.J. (2020). Effects of Teaching Games on Decision Making and Skill Execution: A Systematic Review and Meta-Analysis. Int. J. Environ. Res. Public Health.

[39] Morales-Belando M.T., Kirk D., Arias-Estero J.L. (2022). A Systematic Review of Teaching Games for Understanding Intervention Studies from a Practice-Referenced Perspective. Res. Q. Exerc. Sport.

[40] Liberati A., Altman D.G., Tetzlaff J., Mulrow C., Gøtzsche P.C., Ioannidis J.P.A., Clarke M., Devereaux P.J., Kleijnen J., Moher D. (2009). The PRISMA Statement for Reporting Systematic Reviews and Meta-Analyses of Studies That Evaluate Health Care Interventions: Explanation and Elaboration. PLoS Med..

[41] Page M., McKenzie J., Bossuyt P., Boutron I., Hoffmann T., Mulrow C., Shamseer L., Tetzlaff J., Akl E., Brennan S. (2021). The PRISMA 2020 Statement: An Updated Guideline for Reporting Systematic Reviews. Br. Med. J..

[42] McHugh M.L. (2012). Lessons in Biostatistics Interrater Reliability: The Kappa Statistic. Biochem. Med..

[43] Maher C.G., Sherrington C., Herbert R.D., Moseley A.M., Elkins M. (2003). Reliability of the PEDro Scale for Rating Quality of Randomized. Phys. Ther..

[44] Olosová G., Zapletalová L. (2015). Immediate And Retention Effects Of Teaching Games For Understanding Approach On Basketball Knowledge. Acta Fac. Educ. Phys. Univ. Comen..

[45] Nathan S., Khanna G.L. (2012). A Comparative Approach to Teaching Malaysian School Boys Hockey Using Two Different Pedagogical Styles. Asian J. Exerc. Sport. Sci..

[46] Ioannidis J.P.A. (2008). Interpretation of Tests of Heterogeneity and Bias in Meta-Analysis. J. Eval. Clin. Pract..

[47] Higgins J.P.T., Thompson S.G. (2004). Controlling the Risk of Spurious Findings from Meta-Regression. Stat. Med..

[48] Hastie P.A., Curtner-Smith M. (2006). Influence of a Hybrid Sport Education—Teaching Games for Understanding Unit on One Teacher and His Students. Phys. Educ. Sport Pedagog..

[49] Dominguez S.C., Cobenas E.L., Villaverde A.B., Caballero-Garcia P.A., Sanchez-Paulete N.C., Hernandez-Sampelayo M., Chova L.G., Martinez A.L., Torres I.C. (2013). Visual arts in education. Educational intervention project based learning through contemporary visual arts. Proceedings of the INTED Proceedings, 7th International Technology, Education and Development Conference.

[50] Hassan R., Twyman N.W., Nah F.F.-H., Siau K., Nah F.F.H., Tan C.H. (2016). Patient Engagement in the Medical Facility Waiting Room Using Gamified Healthcare Information Delivery. Proceedings of the HCI in Business, Government, and Organizations: Information Systems, HCIBGO 2016, Held as Part of HCI International 2016.

[51] Arias-Estero J.L., Jaquero P., Martínez-López A.N., Morales-Belando M.T. (2020). Effects of Two TGfU Lessons Period on Game Performance, Knowledge and Psychosocial Variables in Elementary Physical Education. Int. J. Environ. Res. Public Health.

[52] Forrest G. (2015). Systematic Assessment of Game-Centred Approach Practices–the Game-Centred Approach Assessment Scaffold. Phys. Educ. Sport Pedagog..

[53] Griffin L.L., Mitchell S.A., Oslin J.L. (1997). Teaching Sport Concepts and Skills: A Tactical Games Approach.

[54] Barakat M., Doyon J., Debas K., Vandewalle G., Morin A., Poirier G., Martin N., Lafortune M., Karni A., Ungerleider L.G. (2011). Fast and Slow Spindle Involvement in the Consolidation of a New Motor Sequence. Behav. Brain Res..

[55] Ensrud-skraastad O., Haga M. (2020). Associations between Motor Competence, Physical Self-Perception and Autonomous Motivation for Physical Activity in Children. Sports.

[56] Devís J., Sánchez R., Moreno J., Rodríguez P. (1996). La Enseñanza Alternativa de Los Juegos Deportivos: Antecedentes, Modelos Actuales de Iniciación y Reflexiones Finales. Aprendizaje Deportivo.

[57] Jones R.J.A., Marshall S., Peters D.M. (2010). Can We Play a Game Now? The Intrinsic Benefits of TGfU. Eur. J. Phys. Heath Educ..

[58] Sierra-díaz M.J., González-víllora S., Pastor-vicedo J.C. (2019). Can We Motivate Students to Practice Physical Activities and Sports Through Models-Based Practice ? A Systematic Review and Meta-Analysis of Psychosocial Factors Related to Physical Education. Front. Physiol..

[59] Huhtiniemi M., Sääkslahti A., Watt A., Jaakkola T. (2019). Associations among Basic Psychological Needs, Motivation and Enjoyment within Finnish Physical Education Students. J. Sport. Sci. Med..

[60] Mandigo J., Tredway J., Lodewyk K. (2018). Examining the Impact of a Teaching Games for Understanding Approach on the Development of Physical Literacy Using the Passport for Life Assessment Tool. J. Teach. Phys. Educ..

[61] Molina-García J., Queralt A., Estevan I., Sallis J.F. (2016). Ecological Correlates of Spanish Adolescents’ Physical Activity during Physical Education Classes. Eur. Phys. Educ. Rev..

[62] Childs E., de Wit H. (2014). Regular Exercise Is Associated with Emotional Resilience to Acute Stress in Healthy Adults. Front. Physiol..

[63] Alghadir A.H., Gabr S.A., Iqbal Z.A. (2020). Effect of Gender, Physical Activity and Stress-Related Hormones on Adolescent’s Academic Achievements. Int. J. Environ. Res. Public Health.

[64] Hamedinia M., Sharifi M., Hosseini-kakhak A. (2017). The Effect of Eight Weeks of Aerobic, Anaerobic and Resistance Training on Some Factor of Endocannabinoid System, Serotonin, Beta-Endorphin and BDNF in Young Men. Biosci. Biotechnol. Res. Asia.

[65] Collins H., Fawkner S., Booth J., Duncan A.T. (2018). The Effect of Resistance Training Interventions on Weight Status in Youth: A Meta-Analysis. Sport Med..

